# Adipokines in atherosclerosis: unraveling complex roles

**DOI:** 10.3389/fcvm.2023.1235953

**Published:** 2023-08-14

**Authors:** Jiaying Luo, Zhiwei He, Qingwen Li, Mengna Lv, Yuli Cai, Wei Ke, Xuan Niu, Zhaohui Zhang

**Affiliations:** ^1^ Department of Neurology, Renmin Hospital of Wuhan University, Wuhan, China; ^2^ Department of Anesthesiology, Renmin Hospital of Wuhan University, Wuhan, China; ^3^ Department of Endocrinology, Renmin Hospital of Wuhan University, Wuhan, China

**Keywords:** adipokine, atherosclerosis, endothelial cell, vascular smooth muscle cell, macrophage

## Abstract

Adipokines are biologically active factors secreted by adipose tissue that act on local and distant tissues through autocrine, paracrine, and endocrine mechanisms. However, adipokines are believed to be involved in an increased risk of atherosclerosis. Classical adipokines include leptin, adiponectin, and ceramide, while newly identified adipokines include visceral adipose tissue-derived serpin, omentin, and asprosin. New evidence suggests that adipokines can play an essential role in atherosclerosis progression and regression. Here, we summarize the complex roles of various adipokines in atherosclerosis lesions. Representative protective adipokines include adiponectin and neuregulin 4; deteriorating adipokines include leptin, resistin, thrombospondin-1, and C1q/tumor necrosis factor-related protein 5; and adipokines with dual protective and deteriorating effects include C1q/tumor necrosis factor-related protein 1 and C1q/tumor necrosis factor-related protein 3; and adipose tissue-derived bioactive materials include sphingosine-1-phosphate, ceramide, and adipose tissue-derived exosomes. However, the role of a newly discovered adipokine, asprosin, in atherosclerosis remains unclear. This article reviews progress in the research on the effects of adipokines in atherosclerosis and how they may be regulated to halt its progression.

## 1. Introduction

Atherosclerosis (AS) is the leading cause of death from cardiovascular disease (CVD) in Western countries (1). It is characterized by vascular lesion formation, involving dysfunction of vessel wall cells and lipid deposition due to dyslipidemia (2, 3). Obesity, diabetes, and hypertension, which are major cardiovascular risk factors, induce endothelial injury leading to various proatherogenic effects (4). These effects include increased platelet adhesion and aggregation, monocyte adhesion/infiltration, accumulation of oxidatively modified lipoproteins, and vasoconstriction (5). In humans, the initial stage of *de novo* atherosclerotic plaque formation is known as adaptive intimal thickening. It is primarily characterized by the migration and proliferation of vascular smooth muscle cells (VSMCs) (5), during which VSMC transitions from a contracted to a proliferative state. At the late atheroma, VSMCs could migrate to the surface, forming a “fibrous cap” that protects the lesion from rupture (6). Early pathological development is facilitated by VSMC proliferation and migration, while VSMC apoptosis, cellular senescence, and the presence of macrophage-like cells derived from VSMCs may contribute to inflammation (7). The hallmark of AS lesions is the formation of foam cells. Differentiated macrophages express scavenger receptors that recognize and take up oxidized low-density lipoprotein (ox-LDL) (8). Macrophages that accumulate excessive lipids from ox-LDL, transforming into foam cells. In addition to the well-established involvement of lipid accumulation in AS pathogenesis, emerging evidence suggests that adipose tissue, once perceived as a passive energy storage depot, plays an active role in AS by secreting bioactive proteins or products called adipokines (9) (Table 1).

**Table 1:** Vascular components’ role in the development of AS.

Vascular components	Role in development of AS	References
ECs	Persistent shear stress and AS risk factors induce endothelial dysfunction, intimal hyperplasia, and EndMT. ECs express leukocyte adhesion molecules ↑mononuclear phagocytes adhesion.	(10–12)
VSMCs	VSMC senescence is a characteristic of promoting plaque progression and unstable plaques. “Abnormal” proliferation of VSMCs promotes plaque formation. Apoptosis of VSMCs may be the central event of plaque rupture.	(7)
Macrophages	In an atherogenic environment, endothelial adhesion of monocytes is significantly increased, followed by migration to the intima to differentiate into macrophages. Lipoprotein uptake by macrophages through macropinocytosis, phagocytosis, and scavenger receptors (including SR-A, CD 36, and SR-BI) combined with impaired ABCA1 and ABCG1 efflux pathways induces the formation of cholesterol crystals that promote AS lesion progression and necrotic nuclear expansion.	(13, 14)
PVAT	PVAT, under pathological conditions, becomes dysfunctional, secretes pro-inflammatory adipokines, induces endothelial dysfunction and inflammatory cell infiltration, and promotes the development of AS.	(15)

↑ means increase; AS, atherosclerosis; ABCA1, ATP-binding cassette transporter A1; ABCG1, ATP-binding cassette transporter G1; ECs, endothelial cells; EndMT, endothelial-to-mesenchymal transition; PVAT, perivascular adipose tissue; SR-A, type A scavenger receptor; SR-BI, scavenger receptor class B type I; VSMCs, vascular smooth muscle cells.

As one of the largest organs in the body, white adipose tissue (WAT) is mainly composed of adipocytes, blood vessels, lymphocytes, and stem cells, and occupies a central position in energy regulation and metabolism. In the human body, WAT is mainly distributed in subcutaneous, visceral, and gonadal areas. The percentage of WAT in body weight and cell size varies greatly among individuals of different body sizes, from approximately 9%–28% in lean adults to 40%–70% in obese people (16). This disparity primarily arises from the expansion of subcutaneous and visceral WAT depots (17). Notably, there exists considerable heterogeneity in fat cell size, both within an individual and across different individuals. Generally, during periods of weight gain, fat cell size tends to increase, whereas weight loss is associated with a reduction in fat cell size (18). White adipocytes contain large single-compartment lipid droplets with fewer mitochondria, which store excess energy in the body and respond to the body's energy and nutrient needs at all times. Macrophages are the most abundant immune cells in the adipose tissue of obese individuals, and their recruitment and proliferation during high-caloric feeding are usually associated with adipose tissue inflammation and insulin resistance (19, 20). Adipose tissue macrophages in lean organisms tend to be inflammation-ameliorating phenotype, whereas the phenotype of adipose tissue macrophages in obese individuals is more pro-inflammatory (21). The activation of CD8^+^ effector T cells and the recruitment of monocytes and macrophages in obese adipose tissue contribute to the accumulation of adipose tissue macrophages and the promotion of inflammation within the adipose tissue (22). WAT has an endocrine function and secretes characteristic adipokines such as leptin, adiponectin, omentin, and visceral adipose tissue-derived serpin (vaspin). In the obesity model, adipocyte hypertrophy occurred before hyperplasia, with increased secretion of leptin, ceramide, and vaspin and decreased secretion of adiponectin and omentin in hypertrophied adipocytes. One distinguishing feature of hypertrophic WAT is the presence of enlarged adipocytes, which occurs due to lipid accumulation within the cells. In contrast, hyperplastic WAT is characterized by a higher abundance of smaller adipocytes compared to normal or hypertrophic WAT (18). Inducing a brown adipose tissue (BAT) phenotype in WAT is called “browning,” that is, beige adipose tissue (BeAT).

BAT is named for its brown appearance. Color reflects the number of iron-containing mitochondria in adipocytes. The darker the color, the more mitochondria there are (23). In the human body, BAT is mainly found in the interscapular region, back of the neck, and mediastinum. BAT is most abundant in newborns, whereas adults have less than 2% of their body weight in BAT. BAT function decreases with age. Nevertheless, BAT can be restored to its young state when necessary in response to the body's needs (24). Unlike white adipocytes, which are microscopically visible as tiny multi-compartmented lipid droplets, brown adipocytes partly originate from myogenic factor 5 positive progenitor cells and contain more mitochondria than white adipocytes (25). The abundance of mitochondria allows brown adipocytes to achieve adaptive thermogenesis via fatty acid uncoupling and oxidative phosphorylation (26). In addition to this mechanism, alternative pathways for thermogenesis include the succinate cycle, creatine cycle, calcium cycle, fatty acid cycle, and ATP/ADP carrier-mediated thermogenesis (27, 28). Apart from its thermogenic function, BAT and BeAT play a role in metabolic regulation through the secretion of adipokines. Notable adipokines secreted by BAT include neuregulin 4 (NRG4), growth differentiation factor 15 (GDF-15), and fibroblast growth factor 21 (FGF21). While the precise impact of these adipokines on AS remains to be fully elucidated, they hold potential for further exploration in AS research.

Adipose tissue is divided into WAT, BAT and BeAT, the adipocytes of which exhibit different morphological and functional characteristics. BeAT is a reversible state between WAT and BAT and is homologous with WAT,which is considered “browned”WAT (29). Perivascular adipose tissue (PVAT), a connective tissue that envelopes the adventitia of the blood vessels and provide mechanical support, serves as a distinctive form of adipose tissue (30). Perivascular adipose tissue could undergo phenotype change and participate in vascular inflammation and remodeling during atherosclerosis (31). Adipose tissue is now recognized as the largest endocrine organ in the body and a metabolically active organ that plays a vital role in regulating the balance of the systemic energy environment (32). The dysfunction of adipose tissue is directly related to various metabolic diseases, including obesity, CVD, and type 2 diabetes (33, 34). Adipokines are bioactive proteins or products secreted from adipose tissues, and they could exert their effect on local and distant tissues through autocrine, paracrine, and endocrine mechanisms (19, 35). Adipokines could also bind to surface receptors on endothelial cells (ECs), VSMCs, and macrophages, influencing their behavior and modulating AS lesion progression. While adipokines such as adiponectin, NRG4, and C1q/tumor necrosis factor-related protein 9 (CTRP9) have been found to play a protective role in AS, others like leptin, resistin, and thrombospondin-1 (TSP-1) can exacerbate disease progression. Adipokines exert a complex regulatory role in the development of AS. Understanding the beneficial and detrimental effects of specific adipokines can not only serve as biomarkers to predict AS outcomes but also aid in identifying potential therapeutic targets for AS treatment.

**Figure 1:**
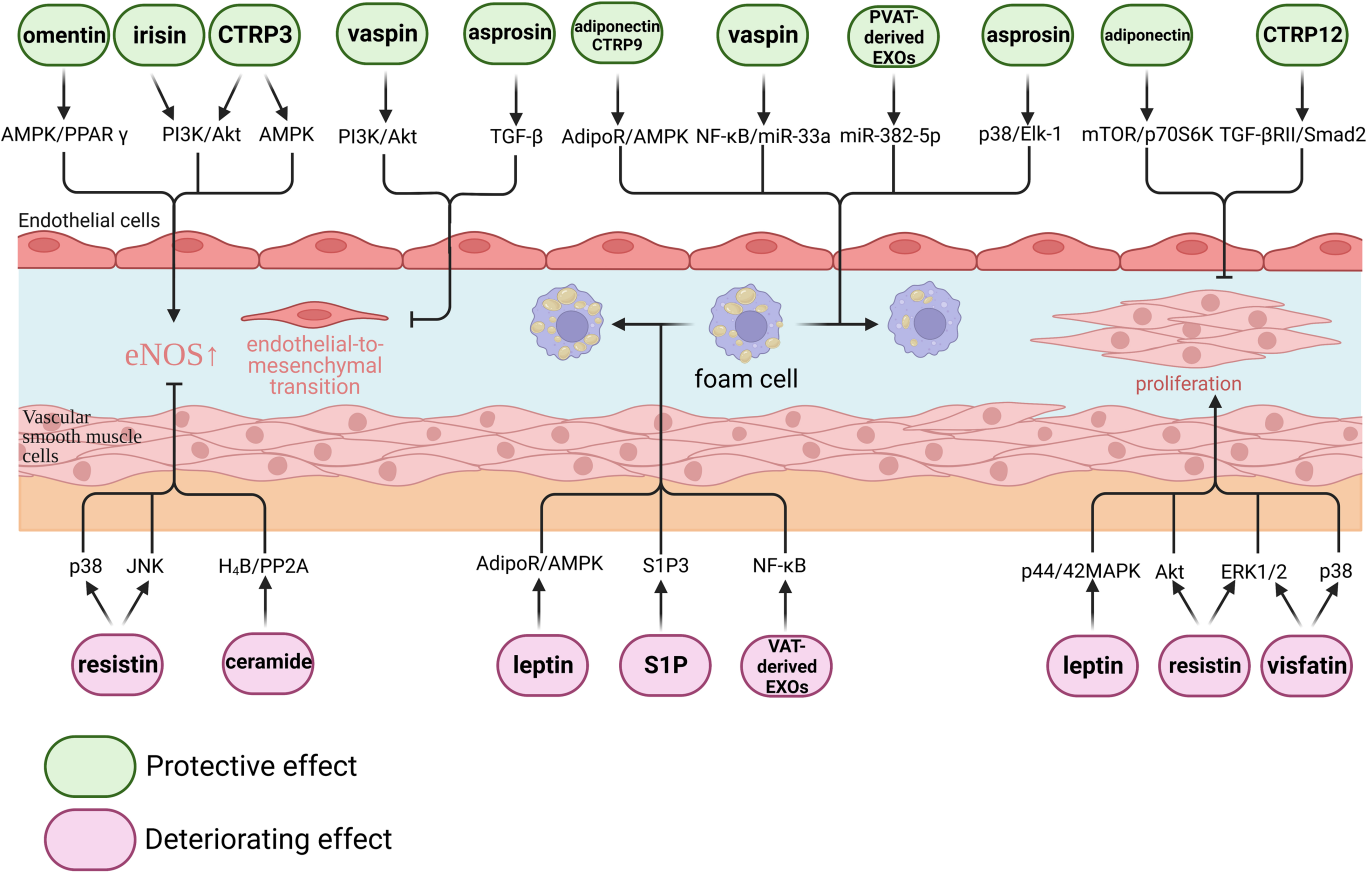
Effect of adipokines on atherosclerosis. Adipokines play complex regulatory roles in atherosclerosis. From a molecular perspective, certain adipokines such as omentin, irisin, CTRP3, vaspin, and asprosin have been shown to exert protective effects on endothelial cells. Conversely, resistin and ceramide have been found to have detrimental effects on endothelial cells. Adiponectin, CTRP9, vaspin, asprosin, and perivascular adipose tissue-derived EXOs have demonstrated the ability to inhibit macrophage foam cell formation. On the other hand, leptin, S1P, and visceral adipose tissue-derived EXOs have been found to promote macrophage foam cell formation. Additionally, adiponectin and CTRP12 have been shown to inhibit vascular smooth muscle cell proliferation, while leptin, resistin, and visfatin have been found to promote vascular smooth muscle cell proliferation.

Adipokines produced by adipose tissue are biologically active. Obese adipose tissue alters the balance of these adipokines and is associated with accelerated CVD. In this review, we classified the role of adipokines in AS progression into five categories: protective, deteriorating, dual-acting, indeterminate and adipose tissue-derived bioactive materials, and summarized the role of these adipokines on AS in Table 2 and Figure 1.

**Table 2:** Adipokines for atherosclerosis.

Classification	Adipokines	Role in the development of AS	References
Anti-atherogenic adipokines	Adiponectin	↓VCAM-1, ICAM-1 expression in ECs, ↑the activity of eNOS ↓proliferation of VSMCs, vascular remodeling, and the activity of iNOS ↑ABCA1/ABCG1 expression in macrophages	(36–39)
	NRG4	↓ECs inflammation and expression of adhesion molecules ↓macrophages aggregation	(33, 40, 41)
	FGF21	↓apoptosis and pyroptosis of ECs ↓calcification of VSMCs ↑ABCA1/ABCG1 expression in macrophages	(42–45)
	Irisin	↑the activity of eNOS ↓osteogenic transition and pyroptosis of VSMCs ↓apoptosis and inflammatory differentiation of macrophages	(46–50)
	SFRP5	↑angiogenesis ↓calcification of VSMCs	(51–53)
Pro-atherogenic adipokines	Leptin	↑ECs inflammation and expression of adhesion molecules ↑proliferation and migration of VSMCs, ↑neointimal formation and vascular remodeling ↑ACAT-1 expression in macrophages	(54–59)
	Resistin	↑central leptin resistance ↑adhesion molecules and inflammatory factors expression in ECs ↑phenotypic switching, proliferation, and migration of VSMCs ↑SR-A/CD36 expression in macrophages	(60–71)
	TSP-1	↓angiogenesis, ↑ECs senescence via the CD47 pathway ↓relaxation of VSMCs via CD36- and CD47-dependent pathways ↑activation of macrophages	(72–78)
	GDF-15	↑ECs senescence ↑chemotaxis of macrophages, ↑lipid accumulation in macrophages	(79–82)
	FABP4	↑ECs inflammation and expression of adhesion molecules ↑proliferation and migration of VSMCs ↑lipid accumulation in macrophages	(83–85)
Indeterminate adipokines	Visfatin	↑ECs senescence via activation of NADPH oxidase, ↑angiogenesis ↑iNOS expression in VSMCs via the ERK1/2 and NF-κB pathway, ↑VSMC proliferation via the ERK1/2 and p38 pathway, ↓vascular remodeling ↑macrophage differentiation	(86–91)
	Omentin	↓ECs apoptosis, ↑the activity of eNOS via the AMPK/PPARδ pathway ↓proliferation and migration of VSMCs, neointimal formation ↓SR-A/CD36 expression in macrophages, ↓atherosclerotic area Circulating omentin level ↑in patients with CAD	(92–95)
	Vaspin	↑ECs NO utilization via the PI3K/Akt pathway, ↓ECs inflammation and EndMT ↓VSMC migration phenotype switching ↓macrophage cholesterol intake via the NF-κB/miR-33a pathway Vaspin is linked to severity of CAD and MACE Gene variants regulate vaspin level, circulating vaspin and subclinical AS markers: no association	(96–105)
	Asprosin	↑ECs EndMT via the TGF-β pathway ↑ABCA1/ABCG1 expression in macrophages via the p38/ELK-1 pathway Circulating asprosin level ↓in patients with carotid plaques and CAD	(106–109)
Dual-acting adipokines	CTRP family	CTRP1 ↓adhesion molecules expression in ECs, ↓vascular remodeling CTRP3 ↑the activity of eNOS via the AMPK pathway, ↓ECs inflammation via the PI3K/Akt/eNOS pathway CTRP5 ↑12/15-LOX in ECs via the STAT6 pathway CTRP9 ↑plaque stabilization, ↓macrophages infiltration in plaque via the AMPK/mTOR pathway ↑autophagy in ECs and macrophages, ↓pro-inflammatory phenotype via the JNK pathway CTRP12 ↓VSMC proliferation via the TGF-βRII/Smad2 pathway ↑anti-inflammatory phenotype via the miR-155-5p/LXRα pathway CTRP12 level is inversely associated with CAD severity, CTRP12 ↑in VAT and SAT of obese subjects	(9, 110–122)
Adipose tissue-derived bioactive materials	Ceramides	↑plaque instabilization ↓ECs NO utilization, ↑uncoupling of eNOS via the H_4_B/PP2A pathway Glucosylceramide ↑plaque vulnerability, ↓cholesterol efflux, circulating glucosylceramide level ↑in patients with CAD Lactosylceramide ↑VSMCs proliferation	(123–128)
	S1P	S1P1 ↑collateral circulation to ischemic brain tissue via eNOS in ECs, ↑cholesterol efflux of atherosclerotic lesions ↑proliferation and migration of VSMCs, ↑neointimal hyperplasia S1P2 ↑ECs inflammation via the NF-κB and the JNK pathways S1P3 ↓macrophages cholesterol efflux	(129–134)
	Exosomes	SAT-derived EXOs: ↑lipolysis in the adipocytes VAT-derived EXOs: ↑pro-inflammatory phenotype by activating NF-κB PVAT-derived EXOs: ↑ABCA1/ABCG1 expression in macrophages via the miR-382-5p and the BMP4-PPARγ pathway	(135–137)

↑ means increase; ↓ means decrease; AS, atherosclerosis; ABCA1, ATP-binding cassette transporter A1; ABCG1, ATP-binding cassette transporter G1; ACAT-1, acyl-coenzyme A cholesterol acyltransferase-1; CAD, coronary artery disease; CTRP, C1q/tumor necrosis factor-related protein; ECs, endothelial cells; EndMT, endothelial-to-mesenchymal transition; eNOS, endothelial nitric oxide synthase; EXOs, exosomes; H_4_B, tetrahydrobiopterin; ICAM-1, intercellular adhesion molecule-1; iNOS, inducible nitric oxide synthase; MACE, major adverse cardiac events; NO, nitric oxide; NRG4, neuregulin 4; PVAT, perivascular adipose tissue; SAT, subcutaneous adipose tissue; SR-A, type A scavenger receptor; SR-BI, scavenger receptor class B type I; S1P, sphingosine-1-phosphate; TSP-1, thrombospondin-1; VAT, visceral adipose tissue; vaspin, visceral adipose tissue-derived serpin; VCAM-1, vascular cellular adhesion molecule-1; VSMCs, vascular smooth muscle cells.

## 2. Protective adipokines for AS

### 2.1. Adiponectin

Adiponectin is a protein-like adipokine secreted by adipocytes (138). It is secreted as multimers of different molecular weights: hexameric, trimeric, and globular. Adiponectin binds to receptors to regulate lipid metabolism and insulin sensitivity. Adiponectin is composed of a globular domain and a collagen-like domain. It interacts with three types of receptors: adiponectin receptors (AdipoRs), calreticulin, and T-cadherin (139). Notably, T-cadherin exhibits high expression levels in the cardiovascular system (140). The binding of the globular domain of adiponectin to T-cadherin has been shown to have beneficial effects on intimal hyperplasia and AS (141). Additionally, the adiponectin/T-cadherin system plays a role in promoting the synthesis of exosomes, small extracellular vesicles, while simultaneously reducing the release of cellular ceramide (142). This dual mechanism contributes to the modulation of insulin sensitivity, thereby potentially mitigating the development of insulin resistance (143). There are two AdipoR types, AdipoR1 and AdipoR2 (144). AdipoR1 and AdipoR2 may have opposite effects on obesity resistance and glucose clearance by activating 5′ AMP-activated protein kinase (AMPK)α1 and AMPKα2, respectively.

Decreased plasma adiponectin levels are associated with an increased risk of intima-media thickness (145) and intracranial AS stenosis (146). Gasbarrino et al. analyzed plasma adiponectin in patients with severe carotid AS undergoing carotid endarterectomy (147). They found that high circulating adiponectin levels were associated with a lower risk of atherosclerotic cardiovascular events. Adiponectin levels are lower in unstable plaques than in stable plaques (148), suggesting that adiponectin levels are negatively associated with the risk of CVD events. Smoking is an essential trigger of AS, and nicotine in tobacco accelerates the progression of AS by reducing adiponectin expression in adipocytes via ATP-dependent potassium channels (149). Physiological concentrations of adiponectin can inhibit the expression of cell adhesion molecules vascular cellular adhesion molecule-1 (VCAM-1), E-selectin, and intercellular adhesion molecule-1 (ICAM-1) on EC (36). Inducible nitric oxide synthase (iNOS) is a nitric oxide (NO) and peroxynitrite-forming enzyme that is overproduced in vascular diseases such as AS or diabetes-related vasculopathy and promotes vascular inflammation and endothelial dysfunction. The globular domain treatment of adiponectin significantly increased endothelial nitric oxide synthase (eNOS) activity but decreased iNOS activity in hyperlipidemic vessels (37), which suggested that adiponectin protects the endothelium from hyperlipidemia through multiple mechanisms. Adiponectin inhibits the mammalian target of rapamycin (mTOR)/p70S6K signaling-mediated proliferation of VSMCs in a receptor-activation-AMPK-dependent (38) or AMPK-independent (39) manner. In addition, adiponectin attenuates angiotensin II–induced vascular remodeling through NO–dependent inhibition of the RhoA/Rho-associated protein kinase pathway and reactive oxygen species production in vascular smooth muscle (150). Adiponectin also regulates lipid efflux in macrophages. Adiponectin reduces lipid accumulation by promoting ATP-binding cassette transporter A1 (ABCA1)- and ATP-binding cassette transporter G1 (ABCG1)-dependent cholesterol efflux through activation of the peroxisome proliferator-activated receptor (PPAR)γ/liver × receptor α signaling pathway (151, 152). Activation of the AdipoR1/AMPK pathway in macrophages reduces apoptosis and improves cholesterol efflux from foam cells, thereby reducing foam cell cholesterol and triglyceride accumulation (153, 154).

### 2.2. Neuregulin 4

NRG4, a novel adipokine, is a member of the NRG family of neuromodulatory proteins. It is mainly expressed in specific peripheral tissues, with the highest expression levels observed in BAT. Li et al. reported that NRG4 could regulate glucose metabolism and improve insulin resistance (155); however, NRG4 has been less studied in AS. NRG4 expression is upregulated in PVAT after vascular injury (33), which mediates ECs to attenuate the expression of inflammatory factors, tumor necrosis factor-α (TNF-α), interleukin-6 (IL-6), and interleukin-1β (IL-1β), and adhesion molecules VCAM-1 and ICAM-1 through the protein Kinase B (Akt)/nuclear factor kappa-B (NF-κB) pathway (40) and inhibits leukocyte migration to the subintima and macrophage accumulation within AS plaques (41). NRG4 also attenuates the levels of inflammatory cytokines in classically activated macrophages (33). The current study suggests that NRG4 inhibits AS development and exerts atheroprotective effects on AS lesions.

### 2.3. FGF21

FGF21 is a signaling protein synthesized in WAT and BAT (156, 157). Exercise can increase plasma levels of FGF21, primarily due to hepatic secretion (158). FGF21 binds to FGF receptors, with the highest affinity for FGF receptors 1 subtype, and β-klotho serves as an essential coreceptor (159). FGF21 has the capacity to reduce fat mass, decrease insulin resistance, and lower plasma glucose and triglyceride levels. Research on FGF21 in adipocytes has primarily focused on human and mouse adipocytes. FGF21 can stimulate glucose uptake in adipocytes by inducing the expression of glucose transporter 1. This process operates independently of insulin and relies on the extracellular regulated protein kinases 1/2 (ERK1/2) signaling pathway and the activation of the serum response factor Ets-like protein 1 (160, 161). FGF21 can stimulate adipocytes to produce and secrete adiponectin through the induction of adiponectin gene expression and PPARγ-dependent mechanisms (162). Additionally, FGF21 can induce browning and adaptive thermogenesis in adipose tissue through various mechanisms, including the induction of PPARγ coactivator 1α and the chemokine C–C motif chemokine ligand 11 (163).

FGF21 exhibits a protective effect on AS. It can inhibit EC apoptosis by suppressing the Fas signaling pathway (42) and inhibit the activation of the NLRP3 inflammasome, thereby preventing EC pyroptosis (43). Aerobic exercise can promote this process by increasing FGF21 levels and downregulating NLRP3 expression (164). Furthermore, FGF21 can promote cholesterol efflux by inducing the expression of ABCA1 and ABCG1 in foam cells and reduce cholesterol accumulation in foam cells through AMPK-mediated autophagy (44, 165). FGF21 also inhibits the calcification of VSMCs (45). These findings suggest that FGF21 may be a potential therapeutic target for preventing and treating AS by modulating EC function, cholesterol metabolism, and vascular calcification.

### 2.4. Irisin

Irisin, an adipomyokine synthesized by skeletal muscle and adipose tissue (166, 167), results from the proteolytic cleavage of membrane-bound FNDC5 (168). It plays a crucial role in regulating energy metabolism and improving insulin resistance by binding to various receptors, including fibroblast growth factor receptors and hemojuvelin (169). Additionally, irisin promotes mitochondrial synthesis and induces browning of WAT (168). Its expression in adipose tissue is decreased in obese individuals (170). Serum irisin levels are significantly lower in patients with coronary artery disease (CAD) and ischemic stroke (171–173), making it a potential predictive marker for early CVDs. Irisin enhances lipid metabolism by facilitating the transport of biliary cholesterol and fecal cholesterol excretion (174). In the experimental model, Irisin-ApoE^−/−^ mice are a strain obtained by crossing Irisin transgenic mice with ApoE^−/−^ mice. Compared to ApoE^−/−^ mice, an improvement in hyperlipidemia was observed in Irisin-ApoE^−/−^ mice, and the irisin levels were negatively correlated with high-density lipoprotein cholesterol (175). Irisin exerts a protective effect against vascular injury and ECs inflammation induced by ox-LDL (176, 177). The protective effect of irisin on ECs is mediated through the activation of the AMPK-PI3K-Akt-eNOS signaling pathway (46). Moreover, irisin reduces macrophage apoptosis induced by ox-LDL, potentially through the inhibition of endoplasmic reticulum stress signaling pathways (47). Irisin also promotes the anti-inflammatory differentiation of macrophages by activating JAK2-STAT6-dependent signaling (48). Additionally, irisin mitigates vascular calcification by suppressing the osteogenic transition and pyroptosis of vascular smooth muscle cells (49, 50). In conclusion, irisin, an adipomyokine with diverse physiological functions, demonstrates significant potential as a biomarker and therapeutic target in various metabolic and CVDs. However, further research is needed to fully elucidate the underlying mechanisms and explore the clinical implications of irisin in human health and disease.

### 2.5. Secreted frizzled-related protein 5

Secreted frizzled-related protein 5 (SFRP5) is an adipokine synthesized by white adipocytes (178). Its expression level is disrupted in obesity under metabolic stress conditions. Studies by Ouchi et al. have demonstrated that SFRP5 expression in adipose tissue is reduced in obesity (179). Functionally, SFRP5 binds to Wnt ligands, thereby interfering with Wnt signaling pathway transduction, which is crucial for promoting adipogenesis (180). Emerging research has consistently shown decreased circulating levels of SFRP5 and increased levels of Wnt5a in patients with CAD and obesity when compared to healthy controls (181, 182). The decrease in circulating SFRP5 levels may serve as an indication that the clearance function of SFRP5 during the obesity stage can still be compensated. By binding to the Wnt ligand Wnt5a, SFRP5 inhibits the activation of the Wnt non-canonical pathway, subsequently promoting inflammation in macrophages and adipose tissue (179).

In the context of vascular health, SFRP5 plays a notable role in inhibiting high phosphate-induced calcification of VSMCs. It achieves this through suppression of the Wnt/β-Catenin pathway and the Wnt3a-mediated signaling (51, 52). In addition to its impact on vascular health, SFRP5 also exhibits angiogenic properties in human umbilical vein ECs by inhibiting the Wnt5a/c-Jun N-terminal kinase (JNK) signaling pathway (53). Furthermore, SFRP5 has been observed to mitigate apoptosis induced by oxidative stress in human aortic ECs (183). The aforementioned findings shed light on the complex involvement of SFRP5 in adipose tissue regulation, metabolic disorders, vascular calcification, and EC function, contributing to our understanding of the intricate interplay between adipokines and physiological processes.

## 3. Deteriorating adipokines for AS

### 3.1. Leptin

Leptin is a pleiotropic hormone secreted by adipocytes, involved in various biological processes, and including inflammatory responses, immune function, and regulation of biological behavior and metabolism. Bäckdahl et al. classified human WAT into three types of mature adipocytes based on distinct transcriptional profiles and spatial arrangement: Adipo*^LEP^*, Adipo*^PLIN^*, and Adipo*^SAA^*. Among these, adipocytes expressing the marker gene *LEP*, which is associated with the synthesis of the adipokine leptin, were categorized as Adipo*^LEP^* (184). Leptin synthesis is regulated by the lipid content in adipocytes, the *Lepob* gene, and adipocyte size (185, 186). There are six subtypes of leptin receptors (LepRa to LepRf) (187), and leptin acts on receptors in the hypothalamus to regulate biological behavior, food intake, and indirectly regulate glucolipid metabolism. Thus, *ob/ob* mice (leptin deficient) and *db/db* mice (leptin receptor deficient) are commonly used models of hyperglycemia and obesity. The same receptors that respond to leptin are also present in the vasculature. In adipose metabolism disorders with reduced serum leptin levels, leptin treatment ameliorates the endothelial-to-mesenchymal transition (188), suggesting an endothelial protective effect of leptin at physiological concentrations.

Central leptin resistance and preservation of peripheral vascular leptin responsiveness allow high leptin concentrations to induce vascular dysfunction (186). A high leptin concentration increases ERK1/2 phosphorylation and NF-κB activation in ECs (54), which leads to increased secretion of the inflammatory factor TNF-α, expression of the cell adhesion molecule VCAM-1 (55), and endothelial leptin resistance (186), disrupting endothelial barrier function. It also exacerbates neointimal growth and vascular remodeling by promoting VSMCs proliferation and metalloproteinase-9 expression that induces migration (56, 57). However, in apolipoprotein E deficient (ApoE^−/−^) mice, leptin-induced neointima formation was entirely blocked (58), indicating that ApoE mediates leptin-induced neointima formation. Additionally, leptin promotes foam cell formation. High leptin concentrations upregulate the expression of acyl-coenzyme A cholesterol acyltransferase-1 in acetylated LDL-induced macrophages, increasing intracellular cholesteryl ester accumulation and promoting foam cell formation (59).

One study demonstrated that the decreased expression of type A scavenger receptor (SR-A) and platelet glycoprotein 4 (CD36) in macrophages of *ob/ob* mice resulted in reduced macrophage cholesterol accumulation and decreased foam cell formation (189). However, another study reported that CD36 expression was upregulated in macrophages of *ob/ob* mice in the presence of defective insulin signaling, leading to increased macrophage foaminess (190). This discrepancy may be because different cellular microenvironments interfere with the macrophage phenotype. In conclusion, high leptin levels can disrupt ECs barrier function, promote VSMCs proliferation and migration, and accelerate AS progression by exacerbating lipid accumulation in foam cells.

### 3.2. Resistin

Resistin belongs to a family of cysteine-rich secreted proteins that are almost exclusively derived from adipose tissue in rodents, with elevated adipose expression and serum levels in models of obesity and insulin resistance (191). Resistin is primarily released by visceral WAT macrophage, exacerbating WAT inflammation (192, 193). Additionally, elevated resistin triggers central leptin resistance (194), exacerbating impaired glucose and lipid metabolism. High resistin expression levels in circulating (60) and unstable plaques (195) in carotid plaque subjects suggest that resistin correlates with carotid disease severity and may serve as a potential marker of plaque instability. Resistin was positively correlated with the degree of thoracic aortic calcification (196) and coronary artery calcification (191), all suggesting a significant association between resistin and the severity of CVD.

Resistin directly contributes to EC activation by promoting the release of the endothelin-1 (197). The major cardiovascular risks contribute to an increase in vascular reactive oxygen species production, which in turn promotes the oxidative degradation of tetrahydrobiopterin, a critical cofactor for eNOS. This process leads to eNOS “uncoupling” and reduced NO production (198). Resistin directly induces eNOS downregulation by excessive ROS production and activation of p38 and JNK in human coronary artery ECs (61). Resistin exacerbates monocyte/macrophage adhesion by stimulating EC upregulation of adhesion molecules VCAM-1 and ICAM-1 via the NF-κB pathway and p38 mitogen-activated protein kinase (MAPK) pathway (62, 63, 199). Additionally, resistin enhances inflammatory factors TNF-α and IL-1β expression via the NF-κB signaling pathway in response to stimulation of human coronary artery ECs (64). PVAT-derived resistin-cultured VSMCs upregulate osteopontin, a hallmark of the phenotype of proliferative VSMCs, via the transcription factor AP-1 (200). Resistin acts on VSMCs in a paracrine or endocrine manner to promote VSMC migration via the protein kinase C protein *ε* pathway (65, 66) and induce human aortic smooth muscle cell proliferation via the ERK 1/2 and Akt signaling pathways (67), respectively. Additionally, protein kinase C protein *ε*-mediated Nox activation induces ROS production, causing VSMC dysfunction and endothelial proliferation (68). In the absence of natural or modified lipoproteins, resistin induces an increase in cholesterol and triglyceride cell mass in human macrophages (201). In contrast, ox-LDL-treated macrophages significantly increased the expression of resistin mRNA (69). Resistin induces a pro-inflammatory phenotype in macrophages via an NF-κB-dependent pathway, increasing macrophage inflammatory factors IL-1, IL-6, IL-12, and TNF-α, as well as VCAM-1, exacerbating vascular inflammation and promoting AS progression (62, 70, 71). Resistin upregulates macrophage SR-A and CD36 via transcription factors AP-1 and PPARγ (69), respectively. Resistin enhances proteasome-mediated degradation of ABCA1, exacerbating cholesterol uptake and facilitating foam cell formation (202). In summary, resistin directly stimulates VSMC proliferation and migration and promotes the inflammatory phenotype of ECs and macrophages.

### 3.3. TSP-1

TSP-1 is a multifunctional glycoprotein secreted by platelets (203), macrophages (204), and adipocytes (205). TSP-1 exerts multiple biological effects by binding to extracellular matrix proteins and cell surface receptors to regulate cell and cell-matrix interactions (206). TSP-1 expression was increased in plasma and visceral adipose tissue (VAT) in both diabetic and obese patients and animals (207). Moreover, TSP-1 expression was greater in VAT than in subcutaneous adipose tissue (SAT) in obese subjects (205). Varma et al. found that it was adipose tissue-derived macrophages rather than THP-1-derived macrophages that expressed TSP-1 (205). Hence, TSP-1 is suggested to be a candidate gene for visceral obesity (208). As an essential ligand of TSP-1, CD36 is also a fatty acid translocase, suggesting that TSP-1 may control the metabolism of fatty acids in adipocytes. Loss of TSP-1 reduced macrophage infiltration in adipose tissue, suggesting that TSP-1 may also regulate inflammatory cells infiltrating expanded adipose tissue.

Not only do CVD risk factors promote TSP-1 expression in adipose tissue, but leptin also promotes TSP-1 synthesis in VSMCs, thus hastening AS progression. High leptin concentrations upregulate TSP-1 expression in VSMCs via JAK2 and MAPK-dependent pathways (209). Furthermore, leptin promotes a synergistic interaction between the transcription factor interferon regulatory factor-1 and the cAMP response element binding protein at the promoter of the TSP-1 gene that drives TSP-1 transcription in VSMCs (210). Moreover, TSP-1 deficiency inhibited leptin-induced VSMC dedifferentiation, lipid loading, and increased plaque area (207), suggesting that the pro-AS effect of leptin is mediated via the TSP-1 pathway.

Diabetes upregulates TSP-1-CD47 signaling in ECs to induce senescence and impair angiogenesis (72). Increased TSP-1 can also interfere with ECs chemotaxis, inhibit ECs proliferation, and capillary formation, thereby inhibiting angiogenesis *in vitro* (73–75). Anti-TSP-1 antibody C6.7 blocks the inhibitory effect of TSP-1 on ECs growth and reendothelialization (211). NO/cGMP signaling enhances intracellular calcium ions and relaxes VSMCs via cGMP-dependent protein kinase (212), while exogenous TSP-1 blocks this diastolic effect of NO via a CD36-dependent pathway (76). Endogenous TSP-1 has the same effect via CD36- and CD47-dependent pathways and also blocks NO-driven ECs adhesion via the CD47 pathway (76, 77). In their study, Moura et al. found that TSP-1 expression increased after carotid ligation and was able to activate VSMCs to induce *de novo* neointima formation (213). In addition, Li et al. found that TSP-1 activates macrophages through a toll-like receptor 4-dependent pathway (78). All of the above suggest that TSP-1 may be a target of AS lesions and play an aggressive role in AS progression.

### 3.4. Growth differentiation factor 15

Growth differentiation factor 15 (GDF-15), a member of the TGFβ superfamily, is expressed in various tissues, including the placenta (214), liver (215), kidney, and brown adipose tissue (216). GDF-15 may play a role in the metabolic dysregulation associated with obesity. Studies have shown that serum levels of GDF-15 are elevated in obese and type 2 diabetes patients compared to lean control groups (217). Similarly, circulating levels of GDF-15 are higher in both obese humans and rodents (218). In the latest research by Sjøberg et al., it was discovered that GDF-15 increases local insulin-stimulated glucose uptake in BAT and WAT through β-adrenergic signaling (219). Notably, GDF-15 expression is strongly induced in BAT in response to cold exposure, while circulating levels of GDF-15 remain unchanged (216). The released GDF-15 from brown adipocytes can target macrophages and downregulate the expression of pro-inflammatory genes through paracrine effects (216). Research has also revealed that mice with GDF-15 deficiency exhibit elevated levels of cholesterol and triglycerides in circulation, and this effect is independent of the *ApoE* gene (220). Moreover, GDF-15 induces anorexia and weight loss through its interaction with the glial-derived neurotrophic factor receptor alpha-like in the central nervous system (221). However, an analysis of the relationship between GDF-15 and weight in non-obese monozygotic twins found a negative correlation between serum levels of GDF-15 and body mass index (217). These findings suggest that the observed increase in GDF-15 in obesity may be a consequence rather than a cause of obesity.

Compared to the normal control group, patients with CAD exhibit significantly elevated circulating levels of GDF-15, suggesting that GDF-15 may serve as an independent predictor of CAD mortality (222). Furthermore, elevated plasma levels of GDF-15 have been identified as an independent predictor of long-term adverse cardiovascular events in patients with moderate CAD (223). These findings indicate that GDF-15 may play a crucial role in the occurrence and progression of CAD. In addition, increased expression of GDF-15 has been observed in atherosclerotic vessels (220), while systemic deficiency of GDF-15 in mice has shown improvements in luminal narrowing in affected vessels (224). Treatment of macrophages with GDF-15 leads to increased levels of autophagy and intracellular lipid accumulation, thereby affecting lipid homeostasis (79). Furthermore, GDF-15 has been shown to exert a pro-inflammatory effect in the progression of AS by mediating the chemotaxis of macrophages through the CCR2 pathway (80). The expression of GDF-15 in ECs is upregulated in response to inflammatory reactions and ROS-mediated cellular senescence (81, 225), and it exerts paracrine effects that impact neighboring non-senescent cells (82). These findings suggest that during vascular stress, the expression of GDF-15 is elevated, and it plays a detrimental role in the progression of AS.

### 3.5. Fatty acid-binding protein 4

Fatty acid-binding protein 4 (FABP4), primarily expressed in adipocytes and macrophages (226). The expression of FABP4 can be reduced through administration of metformin (227) and atorvastatin (228). Under normal conditions, FABP4 is expressed in capillary and venous ECs, but not in arterial ECs (229). However, damaged arteries can induce ectopic expression of FABP4, although the specific receptors involved in this process remain unclear (230). FABP4 has been implicated in AS, and studies using small-molecule inhibitors of aP2 have demonstrated effective treatment for severe AS and type 2 diabetes in mouse models (231).

FABP4 is closely associated with lipid accumulation in monocytes and macrophages. Inhibition of FABP4 can reduce ox-LDL-induced monocyte adhesion by downregulating the expression of integrin β2, integrin α4, and P-selectin glycoprotein ligand 1 (83). Treatment with FABP4 inhibitors in macrophages significantly reduces cholesterol accumulation by increasing the expression of ABCA1 (231). FABP4 promotes inflammatory responses by inhibiting the PPARγ-LXRα-ABCA1 pathway, leading to cholesterol ester accumulation and foam cell formation, and by activating the JNK-AP-1 signaling pathway (84). Metformin reduces lipid accumulation in macrophages by decreasing FOXO1-mediated FABP4 transcription (227). Regarding FABP4 and VSMCs, it has been found that FABP4 can promote migration and proliferation of coronary artery smooth muscle cells through a MAPK-dependent pathway (85). In the context of ECs, inhibition of FABP4 can reduce ox-LDL-induced adhesion of coronary artery ECs by decreasing the expression of ICAM-1, VCAM-1, and P-selectin (83).

Overall, FABP4 plays a significant role in various cellular processes associated with AS, including monocyte adhesion, cholesterol accumulation, foam cell formation, inflammatory responses, and the behavior of smooth muscle and ECs. Inhibition of FABP4 has shown promise as a potential therapeutic strategy for AS and related complications.

## 4. Dual-acting adipokines

### 4.1. CTRP family

C1q/tumor necrosis factor-related proteins (CTRPs) are paralogous homologs of adiponectin. To date, 15 members of this family (CTRP1 to CTRP15) have been identified, exhibiting different or even opposite physiological functions (232). In contrast to adiponectin, which is expressed only in adipocytes, CTRPs are widely distributed *in vivo* and are expressed in the heart, liver, and kidney (232). CTRP1 synthesized in adipose tissue induces proinflammatory and pro-foam cell formation, accelerating AS progression. However, CTRP1 expressed in the vessels has an antithrombotic effect after AS plaque rupture. CTRP1 upregulates ECs adhesion molecule expression via the p38 MAPK/NF-κB pathway (9).

TNF-induced adhesion molecule and cytokine expressions are reduced in ECs and macrophages of CTRP1-deficient mice (9), indicating that CTRP1 induces adhesion molecule expression, inflammatory cytokine production, and promotes leukocyte adhesion to ECs. Moreover, CTRP1 exerts antithrombotic effects by inhibiting collagen-induced platelet agglutination (233). CTRP1 enhances endothelial adhesion molecule-mediated leukocyte homing and accelerates AS progression. CTRP1 overexpression promotes blood monocyte adherence to the vessel wall and their differentiation into macrophages (9). After entering the subendothelium, ox-LDL can induce macrophage CTRP1 expression, leading to enhanced expression of pro-atherogenic inflammatory factors, and PPARγ regulates this effect (234). Systemic administration of an adenoviral vector encoding CTRP1 reduces the growth of human VSMCs via a cAMP-dependent pathway attenuates intimal thickening after vascular injury (110), and prevents pathological vascular remodeling.

In rodent adipose tissue (235), CTRP3 expression is the highest in the mesentery, followed by the epididymis and subcutaneous tissue, and is relatively low in the thorax, perirenal, and BAT. The sources of CTRP3 in adipose tissue are adipocytes, monocytes (236), and fibroblasts (237). CTRP3 is an adipokine with vascular endothelial–protective effects. Decreased CTRP3 expression in epicardial adipose tissue increases the risk of AS in patients with CAD (238). CTRP3 activates the PI3K/Akt/eNOS pathway to attenuate ox-LDL–induced inflammatory responses in mouse aortic ECs (111). The C1q-like globular domain of CTRP3 has been shown to enhance diastolic function in ECs by activating the AMPK/eNOS/NO signaling pathway, thereby preserving vascular endothelial function (112).

CTRP5 is a pro-atherogenic glycoprotein secreted by adipocytes. Adipocyte CTRP5 expression levels in SAT are positively correlated with the degree of obesity in children (239). Serum CTRP5 levels were increased in rodent models of obesity (232). Upregulation of 12/15-lipoxygenase, a key enzyme mediating LDL transport and oxidation in ECs via the signal transducer and activator of transcription 6 signaling pathway, promotes LDL transport across the endothelium and oxidative modifications (113). The globular form of CTRP5 is responsible for diabetic vascular EC dysfunction via Nox1-mediated mitochondrial apoptosis (240). Moreover, CTRP5 can inhibit the expression of uncoupling protein 1, a negative regulator of WAT browning, and cold exposure decreases CTRP5 expression in SAT (241).

The human CTRP9 gene is located on chromosome 13q12.12 (242) and encodes two isoforms, CTRP9A and CTRP9B, whereas mice lack CTRP9B (243). While CTRP9A is secreted as a multimeric protein, CTRP9B requires physical association with CTRP9A or adiponectin for secretion (242). CTRP9 stabilizes plaques and is atheroprotective. Reduced CTRP9 levels are an independent risk factor for CAD in AS patients with thin fibrous caps (114). Defects in the CTRP9 gene alter the gut microbial composition of mice, increase serum cholesterol and LDL levels, and promote AS progression (244). Transplantation of wild-type mice into the intestinal microflora can reverse this effect. Zhang et al. found that CTRP9 exerts atheroprotective effects via the CTRP9-AMPK-NLRP3 inflammatory vesicle pathway (245). CTRP9 promotes EC function and ischemia-induced revascularization through an eNOS-dependent mechanism (246). CTRP9 inhibits EC senescence by promoting autophagy and autophagic flow by activating the AMPK and AMPKα/KLF4 signaling pathways (115, 116). VSMC apoptosis is closely associated with the stability and progression of AS plaques. CTRP9 induces macrophage polarization to the pro-inflammatory phenotype and promotes the apoptosis of VSMCs by activating the JNK pathway (117). CTRP9 overexpression significantly attenuated AS lesion size and reduced the accumulation of macrophages and VSMCs in ApoE^−/−^ mice, which was associated with the activation of the AMPK/mTOR signaling pathway by CTRP9 to induce autophagy (118). CTRP9 also downregulates the inflammatory response of macrophages through the AdipoR1/AMPK pathway, attenuates apoptosis, improves cholesterol efflux from foam cells (153, 245), and improves AS.

CTRP12, also known as adipolin, was found to be a conserved paralog of adiponectin, with the lowest homology to adiponectin among other CTRPs (247). CTRP12 improves insulin sensitivity and glycemic control in obese and diabetic mice (248), with increased expression of CTRP12 in obese VAT and SAT (119). In CAD subjects, serum CTRP12 levels were negatively correlated with the extent of stenosis (120), suggesting that CTRP12 may be an independent protective factor for CAD. CTRP12 reduces the proliferation of VSMCs and inhibits macrophage inflammatory mediator gene expression through the TGF-β receptor II/Smad2-dependent pathway (121, 249). In a study of the effects of CTRP12 on macrophages, it was found that CTRP12 increases ABCA1 and ABCG1-dependent cholesterol efflux and promotes macrophage polarization to the inflammation-ameliorating phenotype via the miR-155-5p/LXRα pathway (122). The above findings suggest that CTRP12, a paralog of adiponectin, may have the same protective role as adiponectin in CVD.

## 5. Indeterminate adipokines

### 5.1. Visfatin

Visfatin is also known as pre-B-cell colony-enhancing factor because it was originally isolated as a secreted factor for synthesizing IL-7 and stem cell factors that promote the growth of B cell precursors. Moreover, visfatin is known as nicotinamide phosphoribosyltransferase (NAMPT), which has enzymatic activity, and intracellular NAMPT is the rate-limiting enzyme that catalyzes the salvage pathway of nicotinamide adenine dinucleotide (NAD+) biosynthesis (250). Extracellular NAMPT, such as adipocyte-derived NAMPT and plasma NAMPT, is enzymatically active and can affect vascular function in an autocrine and paracrine manner (251). Visfatin is mainly released by visceral WAT macrophages (192), while in unstable atherosclerotic lesions, visfatin is expressed by foam cells and macrophages (252). The levels are even higher in subjects with symptomatic carotid plaques, suggesting that visfatin may play a role in unstable plaques.

Visfatin expression is highest in PVAT compared to SAT and VAT. PVAT-derived visfatin stimulates VSMC proliferation through ERK1/2 and p38 signaling pathways (86). iNOS is a NO and peroxynitrite-forming enzyme overproduced in vascular diseases such as AS or diabetes-related vasculopathy and promotes vascular inflammation and endothelial dysfunction. Visfatin induces iNOS expression in VSMCs via ERK1/2- and NF-κB-dependent mechanisms, an effect that can be blocked by the NAMPT inhibitor APO866 (87). Visfatin enhances IL-1β-dependent induction of IL-6 and CD36 production through distinct signaling pathways mediated by JNK and NF-κB, respectively, which leads to accelerated monocyte/macrophage differentiation (88). Activation of NADPH oxidase by visfatin mediates the induction of senescence in human ECs (89). The above findings suggest that visfatin is a cytokine promoting vascular inflammation and AS. However, different from these results, exogenous visfatin ameliorates Ang II-induced endothelial dysfunction and vascular remodeling by targeting NAD/SIRT1 signaling in the study by Zhou et al. (90). Additionally, visfatin can also activate eNOS and improve EC function and angiogenesis *in vitro* and *in vivo* through Akt and MAPK pathways (91). However, it is important to note that these experiments were conducted under conditions of intact endothelial function. Given that endothelial dysfunction is a characteristic feature of AS, further investigations are required to explore the potential beneficial effects of visfatin in the presence of endothelial dysfunction. These inconsistencies between *in vivo* and *in vitro* results still require further studies to explore the underlying mechanisms.

### 5.2. Omentin

Omentin is a relatively new adipokine primarily expressed in VAT (253, 254). There are two highly homologous omentins: omentin-1 and omentin-2. Omentin-1 is the predominant circulating form in human plasma (254). Expression is decreased in metabolic syndromes, such as obesity (255). Elevated levels of omentin-1 in advanced coronary plaques and the circulatory system in patients with acute coronary syndromes may be related to high counteracting AS reactivity (256). Omentin facilitates vasodilation, promotes inflammatory resolution, and inhibits foam cells and neoplastic intima. Compared with ApoE^−/−^ mice, the atherosclerotic area in the aortic sinus of mice expressing the human omentin gene in adipose tissue was significantly reduced, suggesting that omentin may have a protective role in AS (92). At low serum concentrations, omentin promotes the differentiation of human umbilical vein ECs into vascular-like structures to reduce apoptotic activity, activates the AMPK/PPARδ pathway to increase NO production (93), and stimulates ECs to exert vasodilatory physiological effects via an eNOS-dependent mechanism (94), which suggests that circulating omentin concentrations can be a valuable indicator of endothelial function. In addition, omentin-1 regulates macrophage function. Omentin-1 can promote the phosphorylation of Akt in macrophages to exert anti-inflammatory effects and, in turn, promote the conversion of monocytes to anti-inflammatory macrophages *in vitro* and *in vivo* (256), retarding AS development. Omentin inhibits the migration of VSMCs induced by Ang II and platelet-derived growth factor BB (94), decreases matrix metalloproteinase 2 expression after TNF-α stimulation, and significantly inhibits carotid intimal hyperplasia. However, Saely et al. analyzed plasma omentin in patients with coronary angiography and found that increased plasma omentin was a predictor of cardiovascular events in patients with CAD (95). This study's contradictory findings compared to previous conclusions may be attributed to potential racial differences, highlighting the need for further research and investigation. Watanabe et al. found that patients with CAD have decreased omentin levels in the coronary endothelium (256), which may involve a negative feedback regulation mechanism. This opposite result suggests that the role of omentin in the process of AS cannot be fully characterized.

### 5.3. Vaspin

Vaspin is a newly identified adipokine belonging to the serine protease inhibitor family. It is derived from the VAT, and circulating vaspin concentrations are significantly higher in obese vs. lean children (257, 258). Elevated circulating vaspin levels target WAT to exert insulin sensitization and improve glucose tolerance in high-fat diet-induced obese (DIO) rats (259). The long-term injection of vaspin into ApoE^−/−^ mice significantly inhibits the development of AS aortic lesions and increases plaque stability (96). Vaspin is a novel ligand for the GRP78/voltage-dependent negative ion channel complex on the ECs surface (260) and inhibits ECs inflammation by binding to the receptor. Vaspin improves ECs NO utilization through the PI3K/Akt signaling pathway (97, 98) and improves hypoxia-stimulated cell injury and glucose tolerance. Vaspin inhibits acetylcholinesterase in mesenteric arteries to increase acetylcholine-induced eNOS phosphorylation (99). Vaspin inhibits ECs activation induced by the glucose reactive metabolite methylglyoxal (261) and ECs inflammation mediated by proinflammatory factors (100). Serum vaspin levels decrease in patients upon restenosis after coronary stenting. *In vitro* experiments have shown that vaspin inhibits the migration of human coronary artery smooth muscle cells (101), suggesting that it may improve vascular remodeling and prevent stenosis. The osteogenic phenotype switching of VSMCs facilitated vascular calcification. Vaspin eliminates lncRNA LEF1-AS1-mediated VSMCs osteogenic phenotype switching (102) and inhibits the progression of vascular calcification, thereby exacerbating AS. Vaspin inhibits the expression of the receptor for ox-LDL uptake by macrophages via the NF-κB/miR-33a pathway (96), increases cholesterol transport protein expression, and promotes cholesterol efflux, thereby inhibiting the foaming phenotype of macrophages. Decreased circulating vaspin concentrations appear to be associated with CAD severity and a higher incidence of major adverse cardiac events (103, 104). However, Rueda-Gotor et al. did not observe a statistically significant association between vaspin and subclinical AS markers in the study of the relationship between CVD risk and vaspin in patients with axial spondyloarthritis (105). Moreover, they found that serum vaspin concentration was regulated by gene variants (105). Such contradictory results suggest that the role of vaspin in the AS process still requires further investigation.

### 5.4. Asprosin

Asprosin is a newly identified adipokine and a C-terminal cleavage product of pro-fibronectin, primarily expressed in WAT (262). Placental cells, hepatocytes, and cardiomyocytes can also produce this adipokine (263, 264). It plays a vital role in glucose metabolism, appetite regulation, and inflammation; however, little is known about its role in AS (265). Serum asprosin levels are considerably higher in patients with carotid plaques and multiple coronary lesions than in healthy and asymptomatic patients (106, 107, 266). These results suggest that asprosin may be used as a marker for detecting CVD and determining its severity. The expression levels of subcutaneous WAT asprosin are significantly downregulated in mice fed a high-fat diet (265, 267).

Asprosin directly induces the endothelial-to-mesenchymal transition in a TGF-β-dependent manner (108). Asprosin upregulates ABCA1 and ABCG1 expression through activation of the p38/ELK-1 signaling pathway, inhibites lipid expression in THP-1 macrophages during lipid deposition, and reduces AS load in ApoE^−/−^ mice (109). In addition, asprosin negatively regulates WAT browning and enhances lipid accumulation in adipose tissue (267). Based on the previous research findings, the elevated levels of asprosin in the patient's circulation may be a result of negative feedback regulation. However, this conclusion requires further investigation to explore the underlying mechanisms involved.

## 6. Adipose tissue-derived bioactive materials

### 6.1. Ceramides

Ceramide is the precursor of most sphingolipids and the central molecule involved in sphingolipid metabolism. It is also an essential component of cell membranes and a second messenger in critical signaling pathways *in vivo*. Ceramide synthesis occurs in the endoplasmic reticulum (123). There are three pathways for ceramide synthesis *in vivo* (268): *de novo*, sphingolipase, and remedial. In adipocytes, free fatty acids enter the cell and combine with coenzyme A to form acyl-CoAs. In obesity, triglyceride stores in adipocytes become saturated. Excess acyl-CoAs enter the ceramide synthesis pathway, and the concentration of ceramide in the cell increases with free fatty acid accumulation. The adiponectin receptor (AdipoR) has ceramidase activity (269) and works with ceramidase to maintain the dynamic balance between ceramide levels in cells and circulation. Under physiological conditions, ceramide contributes to the regulation of lipid metabolic homeostasis. However, in impaired lipid metabolism, ceramide levels are increased due to the activation of sphingomyelinase, accumulation of fatty acids, and reduced stimulation of AdipoR by downregulating circulating adiponectin levels (270).

Elevated circulating ceramide levels contribute to AS development, insulin resistance, and diabetes mellitus by modulating insulin sensitivity (270), glucose metabolism (271), and lipid metabolism. Increased levels of C16:0, C22:0, and C24:0 ceramides exacerbate the risk of CVDs such as carotid plaque and stroke (272). Reducing ceramide synthesis in adipocytes slows AS development. Ceramide induces uncoupling of eNOS in ECs via the tetrahydrobiopterin/protein phosphatase 2 pathway, decreases NO utilization (124), blocks vasodilation, and increases the risk of CVD. Simultaneously, ceramide promotes apoptosis of VSMCs (123) and the development of unstable plaques. The expression and secretion of glucosylceramide and lactosylceramide increase in adipocytes when adipose tissue metabolism is impaired. Human aortic AS plaque analysis revealed high glucosylceramide and lactosylceramide levels (125). Fiorelli et al. found increased expression of lactosylceramide in monocytes from patients with acute myocardial infarction (126). In a plasma lipid profile analysis of 200 patients with carotid plaques, high circulating glucosylceramide levels increased plaque vulnerability (273). Lactosylceramide also stimulates VSMC proliferation by stimulating nuclear antigen expression, promoting aortic VSMC proliferation (127), and facilitating monocyte migration (126). In ApoE^−/−^ mice, inhibition of glucosylceramide synthesis promotes cholesterol excretion (274) and reduces AS plaque load.

### 6.2. S1P

S1P is a bioactive sphingolipid produced by the phosphorylation of sphingosine and is catalyzed by two sphingosine kinase isozymes (SphK1 and SphK2). S1P triggers inflammation by interacting with five different receptor types, S1P1–5, which belong to the G-protein-coupled receptor family. The major S1P receptors in the vascular system are S1P2, S1P1, and S1P3. The major carrier proteins of S1P are apolipoprotein M (ApoM) and albumin. The effect of S1P on the inflammatory response depends on its carrier protein. Most plasma S1P binds to ApoM to form the ApoM-S1P complex, which preferentially binds to high-density lipoprotein (HDL) and activates S1P1 (270). Although it is a product of the same sphingomyelin as ceramide, S1P has a different physiological function. Unlike S1P2, which aggravates endothelial impairment (129), S1P1 and S1P3 safeguard ECs barrier function and promote vasodilation to improve the tissue blood supply (130, 275, 276). S1P1 is expressed in cerebrovascular ECs and, upon activation, maintains responsiveness to vasodilatory stimuli, and may ensure collateral circulation to ischemic brain tissue via eNOS (130). Apolipoprotein M and S1P1 promote transcytosis of HDL across endothelial monolayers and induce cholesterol efflux from AS lesions to reduce the plaque lipid load (131). S1P2 promotes ECs expression of inflammatory factors and cell adhesion molecules by activating NF-κB (132) and JNK phosphorylation pathways (129). S1P2 also induces senescence-associated damage in young ECs (277). Under hypoxic conditions, S1P3 is activated in ECs, resulting in NO-dependent vasodilation (278).

S1P1 and S1P2 play different roles in VSMCs proliferation and migration. After ligation of the carotid arteries in mice, S1P1 expression is upregulated in the carotid arteries. After vascular injury, the S1P-S1P1 signaling pathway promotes VSMCs proliferation and migration and promotes neointimal hyperplasia (133). In contrast, S1P2 can inhibit S1P-induced migration of human coronary artery smooth muscle cells via HDL (279). S1P1 can also promote the conversion of macrophages to an anti-inflammatory phenotype (280). High endogenous S1P levels activate S1P3, impair macrophage cholesterol efflux, and cause plaque rupture (134). S1P4 is expressed in primary brain microvascular ECs, and S1P4 expression decreases after stroke (281); S1P5 reduces activation of the transcription factor NF-κB and maintains low expression levels of leukocyte adhesion molecules, inflammatory chemokines, and cytokines (282). Both S1P4 and S1P5 in brain ECs have strong protective barriers.

### 6.3. Exosomes

After the fusion of the intracellular body of multivesicular vesicles with the plasma membrane of the parent cell, the inner vesicles inside are expelled from the cell, forming exosomes (EXOs) (283). EXOs can affect the composition of the extracellular matrix and mediate intercellular communication and information transfer after cellular excretion (284). EXOs differ by secretory tissue, resulting in heterogeneity in size, composition, and cellular or organ uptake (285). WAT-derived EXOs are pro-atherogenic in obesity. In high-fat DIO mice, EXOs from SAT caused changes in lipid profiles (135). EXOs extracted from SAT of DIO mice aggravated obesity by affecting fatty acid metabolism in mice, while EXOs from VAT promoted macrophage foaminess and converted them to the pro-inflammatory phenotype by activating NF-κB (136). PVAT-derived EXOs can reduce macrophage foaminess through the miR-382-5p- and bone morphogenetic protein 4-PPARγ-mediated upregulation of the cholesterol efflux transporters ABCA1 and ABCG1. In contrast, this effect is weakened in CAD patients due to the downregulation of miR-382-5p expression (137). EXOs secreted by adipose-derived mesenchymal stem cells reduce reactive oxygen species production in ECs, promote angiogenesis (286, 287), and induce inflammation-ameliorating phenotype polarization of macrophages (288). In addition, ECs-derived (289) and VSMCs-derived (290) EXOs interact with each other, forming a new mode of cell-cell communication. Cytokine-stimulated VSMCs-derived EXOs cause VSMCs self-malfunction/proliferation and induce ECs malfunction, which can be attenuated by the miR548ai inhibitor (290). ECs-derived EXOs, in turn, enhance leukocyte adhesion to VSMCs and induce VSMCs protein synthesis and senescence (291). Exosomal lncRNA LIPCAR derived from THP-1 cells modified by ox-LDL significantly increases the expression levels of cyclin dependent kinase 2 and proliferating cell nuclear antigen in human vascular VSMCs to promote AS progression (292). Activated platelet-derived EXOs (293) decreases macrophage CD36 content, attenuates the CD36-dependent lipid loading capacity of macrophages, and inhibites platelet aggregation and thrombosis.

## 7. Conclusions

The increasing number of overweight and obese individuals has sparked interest in the role of adipose tissue in inducing associated comorbidities. Adipokines have been extensively studied due to their multifactorial properties in regulating physiological functions. Leptin, adiponectin, resistin, and visfatin have been extensively studied and play important roles in regulating glucose metabolism and cardiovascular homeostasis. Additionally, deteriorative adipokines contribute to metabolic dysregulation, endothelial dysfunction, vascular remodeling, and foam cell formation, leading to AS and an increased risk of CVD. The role of some adipokines in the pathogenesis of AS has been established. However, adipokines with unclear roles, such as omentin, vaspin, etc., still require larger prospective studies in the general population and patients with CVD to determine whether measuring circulating levels of adipokines improves AS prediction. Nevertheless, the functions of adipokines are coordinated, and changes in one adipokine may affect others. Adipokines may represent a novel clinical approach to reduce CVD-related mortality and disability, but only positive and highly effective results from well-designed clinical trials will allow broad therapeutic intervention targeting circulating adipokines.

In recent years, various adipokines have been gradually recognized has research progressed, and their role in the mechanism of AS has been explored. On the one hand, the representative protective adipokines, such as adiponectin, omentin, CTRP9, and vaspin, mainly prevent AS by protecting ECs function, inhibiting VSMCs proliferation and migration, and reducing macrophage inflammation and foam cell formation. However, their specific regulatory mechanisms differ from each other. For example, adiponectin inhibits mTOR/p70S6K signaling (38, 39) to attenuate VSMCs proliferation, whereas CTRP9 promotes VSMCs apoptosis by activating the JNK pathway (117). Asprosin ameliorates macrophage cholesterol efflux by activating the p38/ELK-1 signaling pathway to upregulate ABCA1 and ABCG1 expression (109), whereas CTRP9 improves macrophage cholesterol efflux via the AdipoR1/AMPK pathway (153). Similarly, to increase NO utilization by ECs, omentin activates the AMPK/PPARδ pathway (93), whereas vaspin activates the PI3K/Akt signaling pathway (98).

In contrast, ceramide, leptin, and CTRP5 are risk factors for AS, inducing eNOS uncoupling in ECs, promoting neointima formation, and accelerating macrophage foam cell formation. Leptin and CTRP5 have different regulatory mechanisms in ECs. High concentrations of leptin increase phosphorylation of ERK1/2 and activate NF-κB in ECs, leading to increased expression of inflammatory factors and cell adhesion molecules, and worsening endothelial injury (54). CTRP5 promotes LDL transcytosis transport and oxidative damage by activating the signal transducer and activator of transcription 6 signaling pathway to upregulate 12/15-lipoxygenase (113), a key enzyme mediating LDL transport and oxidation in ECs.

Some adipokines and adipose tissue-derived bioactive materials, such as S1P, CTRP1, and CTRP3, have both positive and negative effects on AS lesions. The inconsistency in the roles played by different cell types or ligands in the progression of AS after activation has revealed various roles played by these adipokines in AS. The roles of EXOs secreted by different adipose tissues vary, resulting in different effects on the same cells, forming the basis for the diverse functions of EXOs. Finally, the role of adipokine asprosin in AS lesions is uncertain. Due to the limited research on asprosin in vascular lesions, no conclusions can be drawn, and further investigation is warranted.

In conclusion, adipokines play a complex regulatory role in the development of AS. Increasing our understanding of which adipokines are beneficial or detrimental will help predict atherogenesis biomarkers and help identify potential therapeutic targets for AS.
